# Rumination Mediates the Relation of Hostile Attribution to Psychological Maladjustment Among Adolescents from Three Countries

**DOI:** 10.1007/s10802-025-01288-z

**Published:** 2025-01-31

**Authors:** Anne-Marie R. Iselin, Jamie DeCoster, Laura DiGiunta, Jennifer E. Lansford, Kenneth A. Dodge, Nancy Eisenberg, Concetta Pastorelli, Liliana Maria Uribe Tirado, Dario Bacchini

**Affiliations:** 1 Psychology Department, Elon University, Elon, USA; 2 Center for Advanced Study of Teaching & Learning, University of Virginia, Charlottesville, USA; 3 Department of Psychology, Sapienza University of Rome, Rome, Italy; 4 Center for Child and Family Policy, Duke University, Durham, USA; 5 Psychology Department, Arizona State University, Tempe, USA; 6 Psychology Department, Universidad San Buenaventura, Bogotá, Colombia; 7 Psychology Department, Federico II Second University of Naples, Naples, Italy; 8 Elon University, 2337 Campus Box, Elon, NC 27244-2010 USA

**Keywords:** Attribution bias, Rumination, Aggression, Anxiety

## Abstract

Addressing global concerns about youth mental health requires understanding longitudinal pathways to psychological maladjustment among diverse youth. Hostile attribution bias (HAB) and hostile rumination (HR) are cognitive vulnerabilities associated with multiple forms of psychological maladjustment among diverse youth. This study longitudinally examined whether HR mediates the relation of HAB to aggression, anxiety, and depression symptoms in a sample of adolescents from three countries. Participants included 532 mothers, 384 fathers, and 566 youth (50% female) from Colombia, Italy (Naples and Rome), and the U.S. (White, Black, and Latinx). Structural equation modeling indicated that youth-reported HR at Time 2 (mean age = 12.58 years) significantly mediated the relation of youth-reported HAB at Time 1 (mean age = 10.89 years) to parent-reported aggression and anxiety symptoms at Time 3 (mean age = 13.71 years; aggression: *b* = 0.05, 95% bootstrap CI = [0.006, 0.14]; anxiety: *b* = 0.06, 95% bootstrap CI = [0.01, 0.16]); but not to parent-reported depression symptoms at Time 3 (*b* = 0.02, 95% bootstrap CI = [-0.04, 0.08]). A reverse model indicated HAB at Time 3 significantly mediated the relation of HR at Time 2 to anxiety symptoms at Time 4 (mean age = 14.99 years; *b* = -0.01, 95% bootstrap CI = [-0.04, -0.001]), but not to aggression or depression symptoms at Time 4. Multi-group analyses indicated focal mediational paths did not vary significantly across national, regional, and racial subgroups or gender. Findings support the Integrative Cognitive Model of Aggression, providing evidence that HAB and HR may be vulnerability factors for aggression and anxiety among diverse youth.

**Supplementary Information:**

The online version contains supplementary material available at 10.1007/s10802-025-01288-z.

Psychological maladjustment in childhood and adolescence can adversely affect health and wellbeing in adulthood (Oerlemans et al., 2020). It is therefore imperative to identify longitudinal pathways to psychological maladjustment because many individuals with persistent mental health concerns in adulthood began experiencing symptoms in adolescence (Kessler et al., 2005). One potential pathway predicting psychological maladjustment might involve cognitive vulnerability factors such as negative interpretation biases and ruminative response styles. Research suggests these mechanisms are associated with multiple forms of psychological maladjustment; thus, a better understanding of negative interpretation biases and ruminative response styles could expand the reach and applicability of evidence-based therapeutic practices (Fraire & Ollendick, 2013; Platt et al., 2017). The current study used a longitudinal design to test pathways through two transdiagnostic cognitive vulnerability factors—hostile attribution bias (HAB; interpreting ambiguous social provocations as threatening) and hostile rumination (HR; repetitively dwelling on threatening aspects of a social exchange)—to aggression, anxiety, and depression symptoms among nationally, regionally, and racially diverse youth from three countries.

## Hostile Attribution Bias and Psychological Maladjustment

Negative cognitive interpretation biases are personal tendencies to make negative inferences about ambiguous social provocations (Bӧgels et al., 2003). Systematic reviews indicate negative interpretation biases^1^ are associated with an array of psychological difficulties, including aggression, anxiety, and depression (Platt et al., 2017; Stuijfzand et al., 2018; Verhoef et al., 2019). HAB is a specific negative interpretation bias that has been associated with several maladaptive psychological outcomes, including aggression, depression, and anxiety. A meta-analysis of 219 effect sizes relating HAB to aggressive behaviors among children found a small-to-moderate mean effect size (*d* = 0.33) with no significant difference in effect sizes when comparing reactive aggression with general aggression (Verhoef et al., 2019). Investigations of intraindividual changes also link HAB to aggression. Based on weekly diary reports across four weeks, Alsem et al. (2022) found aggressive behaviors increased in weeks when there were also increases in HAB. Furthermore, treatments reducing HABs have led to decreases in aggressive behaviors (Dodge et al., 2013; Hudley & Graham, 1993; Sukhodolsky et al., 2005; Vassilopoulos et al., 2014; for an exception, see Hiemstra et al., 2019). Existing empirical evidence indicates the relation of HAB with aggression is robust in size and might be temporally ordered with HAB leading to aggression.

Although aggression has been the most frequently examined consequence of HAB, HAB is related to other outcomes as well (Dodge, 2006). Anxious youth incorrectly interpret benign situations as hostile more frequently than non-anxious youth (Bell-Dolan, 1995). However, the relation of HAB with anxiety symptoms might be inconsistent because Alsem et al. (2023) found evidence for this relation in only one of two studies. Beyond anxiety, HAB has been associated with depression among youth (Quiggle et al., 1992).

Intervention studies temporally link negative interpretation biases (not including HAB) to anxiety and depression. A meta-analysis of 67 randomized controlled studies found that reductions in negative and/or increases in positive interpretation biases lead to reduced anxiety and depression symptoms, with effect sizes ranging from small (*g* = 0.32) to large (*g* = 0.71; Martinelli et al., 2022). This meta-analysis included studies of youth and adults; however, age was not a significant moderator of effect sizes. Martinelli et al. also found that effect sizes were larger in non-clinical than clinical samples. Evidence to date implicates HAB as an important cognitive risk factor for aggression and suggests it also predicts anxiety and depression symptoms.

## Relating HAB and HR to Psychological Maladjustment

Caprara et al. (2007) theorized that youth who frequently interpret ambiguous social provocations as hostile are more likely to engage in HR. Repetitively dwelling on hostile stimuli can amplify and prolong antagonistic feelings, increasing the likelihood youth will act in ways consistent with those feelings (Caprara et al., 2007). Following from Crick and Dodge (1994), Dodge et al. (2022) articulated and empirically supported a sequence of social-information-processing steps that individuals follow in response to a social stimulus and a particular sequence that leads to behavioral maladjustment. This sequence starts with selective attention to negative cues and moves to HAB, which triggers emotional responses that include HR. From there, additional mental processes lead to dysfunctional behavior. Thus, it is reasonable to hypothesize that HAB will predict HR, which in turn predicts psychological maladjustment.

The Integrative Cognitive Model of Aggression posits a mediational pathway from HAB through HR to aggression (Wilkowski & Robinson, 2008). In research with young adults, Quan et al. (2019) found a significant indirect effect of HAB on aggression through HR (providing statistical support for mediation). Similarly, Wang et al. (2019) found that HAB precedes HR when explaining aggressive behaviors (verifying the temporal order for mediation). Based on these studies with young adults, it is plausible that HR temporally follows HAB and could mediate the relation of HAB to aggression among youth.

Prior research and theory support the hypothesis that HR mediates the relation of HAB to aggression among youth. As previously mentioned, Caprara et al. (2007) theoretically proposed that HAB underpins hostile rumination (HR) among youth. Second, heightened HR was associated with aggression among youth (Harmon et al., 2019; Smith et al., 2016), and higher HR across adolescence predicted engaging in more aggressive and violent behaviors in young adulthood (Caprara et al., 2007). Thus, the relation of HAB to HR is theoretically supported and the relation of HR to aggression is empirically supported; however, a full mediation model examining whether HR explains the HAB to aggression pathway has not been examined among adolescents. It is important to examine whether such a mediational pathway is evident earlier in life (i.e., adolescence) when processes such as HAB and HR are more amenable to change in ways that decrease aggression and increase adaptive adjustment (Dodge, 2006).

The Integrative Cognitive Model of Aggression focuses only on explaining anger and aggression. However, other psychological maladjustment outcomes are likely to follow from the influence of HAB on HR. First, as previously noted, HABs are related to anxiety and depression symptoms (Barrett et al., 1996; Quiggle, 1992). Clinical theories indicate that negative interpretation biases (e.g., The Generic Cognitive Model; Beck & Haigh, 2014) and anger (Cassiello-Robbins & Barlow, 2016) play important roles in the development of internalizing symptoms. HAB might therefore link these two theoretical domains to explain increases in anxiety and depressive symptoms. Second, rumination, conceptualized broadly, is transdiagnostic (Nolen-Hoeksema & Watkins, 2011). Meta-analyses indicate large relations of rumination with depressive (*r* =.51) and anxiety symptoms (*r* =.46) among youths (Schäfer et al., 2017). There is also preliminary evidence that HR may be transdiagnostic, predicting both aggression and depressive symptoms among preadolescent children (Harmon et al., 2019). To our knowledge, research has yet to link HR to anxiety symptoms. Additional evidence on whether HR is transdiagnostic could expand the Integrative Cognitive Model of Aggression to include anxiety and depressive symptoms. Examining multiple forms of psychological maladjustment extends the reach and applicability of the Integrative Cognitive Model of Aggression, which in turn may expand the reach of evidence-based therapeutic practices based on the theory.

Within Dodge’s (2006) theoretical framework, it is also plausible that the emergence of HAB is through HR. If HR becomes a chronic knowledge structure of a child, it could guide one’s attentional focus on future hostile cues. Engaging in HR over time could generate a perceptual readiness that facilitates access to hostile information in subsequent social interactions and could lead to a tendency to make hostile attributions about future cues. It is therefore important to examine the plausibility of whether HR increases one’s tendency to engage in HAB, which in turn contributes to maladaptive outcomes such as aggression, depression, and anxiety symptoms. We test this plausible alternative mediational pathway from HR to HAB to psychological maladjustment and note these two models are not incompatible.

## Application to Samples of Diverse Youth

Several studies have examined relations among HAB, HR, aggression, anxiety, and depression symptoms in diverse samples of youth from countries around the world. For instance, the positive relation of HAB to aggression replicates across globally diverse community samples of children from nine different countries (some of which are included in the current study; Dodge et al., 2015). HR has been positively associated with aggression among youth from different countries (e.g., youth from Italy in Caprara et al., 2007; youth from the U.S. in Harmon et al., 2019). These studies provide preliminary evidence supporting pathways from both HAB and HR to aggression among diverse samples of youth; however, mediational pathways (e.g., HAB through HR to aggression) have not yet been examined.

Whether pathways from HAB through HR to anxiety and depression symptoms function similarly across diverse youth is less clear. HAB has been associated with anxiety among youth in the U.S. (Bell-Dolan, 1995) and the Netherlands (Alsem et al., 2023). Furthermore, HR has been associated with depressive symptoms among youth in the U.S. (Harmon et al., 2019), but not among youth in Greece (Spyropoulou & Giovazolias, 2022). Examining whether HR mediates the relation of HAB to anxiety and depression symptoms in samples from multiple countries and racial groups could help clarify whether HR functions as a transdiagnostic mediator among diverse youth.

## The Current Study

The Integrative Cognitive Model of Aggression (Wilkowski & Robinson, 2008) posits that HR can explain the relation of HAB to aggression, although evidence supporting this mediational pathway is limited to adult samples. Preliminary evidence from youth supports the relations of HAB to HR and of HR to psychological maladjustment, yet full mediational models have not been examined among youth. Examining these relations among adolescents could inform evidence-based practices to reduce aggression, anxiety, and depression by preventing HAB and HR tendencies from persisting into adulthood when they are more difficult to change (Dodge, 2006). Evidence from nationally and racially diverse individuals is also needed so prevention and intervention efforts seeking to enhance healthy adaptation are optimal for youth from a range of diverse backgrounds.

Using longitudinal data from nationally and racially diverse early adolescents, the current study investigated multiple pathways to psychological maladjustment. We first tested the hypothesis that HR mediates the relation of HAB to aggression, anxiety, and depression symptoms. We then tested the alternate hypothesis that HAB mediates the relation of HR to aggression, anxiety, and depression symptoms. Finally, we tested the proposition that the mediational pathways we examined were consistent across national, regional, and racial subgroups of youth as well as across genders.

## Method

### Participants

Participants were mothers, fathers, and children from three countries—Colombia, Italy, and the U.S.—enrolled in the Parenting Across Cultures Study (e.g., Lansford et al., 2014). Data collection occurred in four geographic regions: Medellín, Colombia; Naples and Rome, Italy; and Durham, North Carolina, U.S. Within the U.S., we analyzed data from three racial/ethnic groups: White, Black, and Latinx. We sought samples from countries/regions varying across a range of socio-demographic characteristics, including race, ethnicity, religion, parental education levels/socioeconomic status, and child well-being indicators. Our three countries varied on their Health Development Index score (HDI; i.e., a composite indicator that ranks countries based on health, education, and income). For example, based on 2012 HDI data (aligned with the middle time points in our data), the U.S. ranked 5th, Italy 28th, and Colombia 98th out of 187 countries. We also sought samples within the U.S. that varied on socio-historical and cultural experiences (e.g., differences in exposure to environmental stressors, experiences of discrimination) which could influence our variables of interest^2^. Despite being quite diverse, participants came from convenience samples and are therefore not representative of entire cultures, nations, regions, or racial/ethnic groups.

Our total sample included 532 mothers, 384 fathers, and 566 children (siblings excluded; 50% girls). Sample sizes across subgroups were: Medellín = 100 adolescents, 85 mothers, 74 fathers; Naples = 95 adolescents, 94 mothers, 73 fathers; Rome = 99 adolescents, 99 mothers, 77 fathers; U.S. White = 100 adolescents, 95 mothers, 67 fathers; U.S. Black = 92 adolescents, 87 mothers, 50 fathers; and U.S. Latinx = 80 adolescents, 72 mothers, 43 fathers. Detailed socio-demographic information about families within each subgroup is presented in Supplemental Materials Table S1and in Iselin et al. (2022). In short, there was variability across the six subgroups in marital status (e.g., 37–83% married parents), parent education level (e.g., 10 to 16 years, on average), and percent working mothers (42–78%).

We analyzed data gathered at four time points [T1: youth *Mage* = 10.89 years (*SD* = 0.70); T2: youth *Mage* = 12.58 years (*SD* = 0.68); T3: youth *Mage* = 13.71 years (*SD* = 0.67); T4: youth *Mage* = 14.99 years (*SD* = 0.73)]. Youth-reported HAB was assessed at T1 and T3, youth-reported HR at T2, and parent-reported aggression, anxiety, and depression symptoms at T1, T2, T3, and T4. The overall retention rate was over 95% within the full sample across all time points. T1, T2, T3, and T4 data were gathered in 2010–2011, 2012–2013, 2012–2014, and 2014–2016, respectively.

### Measures

#### Hostile Attribution Bias (T1 and T3)

On this measure, youth imagined themselves experiencing hypothetical social interactions that involved ambiguous peer provocations (e.g., wearing new shoes and being bumped into a puddle; see Dodge et al., 2015, for more details). For each of 10 different vignettes, youth rated whether the provocateur engaged in the behavior on accident (scored 0) or on purpose (scored 1). We averaged scores across items to create a composite measure of HAB. Providing evidence for the validity and utility of this measure, Dodge et al. (2015) found that this measure of HAB predicted both child- and parent-reported aggression among children from several different countries including those investigated in the current study. In the current study, reliability (i.e., Cronbach’s alpha, McDonald’s omega) was slightly below levels typically identified as adequate (approximately 0.60; see Table 1). Low reliability makes it more difficult to find significant relations but will not lead to spuriously significant results (Cohen et al., 2003). We should therefore be cautious about interpreting nonsignificant relations with HAB but can interpret any significant relations we observe.

**Table 1:** Descriptive statistics for theoretical variables

	*N*	Mean	Scale range^a^	SD	Cronbach’s α reliability	McDonald’s ω reliability
T1 Age	567	10.89	--	0.70	--	--
T1 Aggression	566	0.34	0 to 2	0.28	0.89	0.90
T1 Depression	566	0.21	0 to 2	0.26	0.73	0.74
T1 Anxiety	566	0.60	0 to 2	0.39	0.78	0.78
T1 HAB	566	0.43	0 to 1	0.23	0.63	0.63
T2 HR	543	2.32	0 to 5	0.98	0.71	0.71
T2 Aggression	549	0.34	0 to 2	0.31	0.88	0.89
T2 Depression	549	0.22	0 to 2	0.31	0.70	0.72
T2 Anxiety	549	0.57	0 to 2	0.45	0.75	0.75
T3 HAB	534	0.39	0 to 1	0.21	0.57	0.60
T3 Aggression	538	0.32	0 to 2	0.26	0.88	0.89
T3 Depression	538	0.20	0 to 2	0.25	0.73	0.73
T3 Anxiety	538	0.56	0 to 2	0.39	0.77	0.77
T4 Aggression	517	0.30	0 to 2	0.27	0.89	0.89
T4 Depression	517	0.20	0 to 2	0.28	0.79	0.80
T4 Anxiety	517	0.54	0 to 2	0.40	0.79	0.79

*Note *^a^ = for all scales, higher scores indicate higher maladaptive thoughts, feelings, and behaviors

#### Hostile Rumination (T2)

The Hostile Rumination Scale (Caprara, 1986) asks youth to report how characteristic (0 = *completely false* to 5 = *completely true*) it is for them to brood on feelings of anger and resentment and to want retribution. To create a composite measure of HR, we averaged scores across seven items, dropping one item based on Iselin et al. (2022). Iselin et al. (2022) also provide evidence of measurement invariance across subgroups included in this study, and additional research provides evidence supporting the validity of HR across multiple countries, regions and racial groups included in this study (e.g., Di Giunta et al., 2017). Furthermore, Caprara et al. (2007) and Caprara et al. (2017) found that the measure of HR used in this study was significantly related to irritability, aggression, and violence among youth. Reliability was adequate in the current study (see Table 1).

#### Aggression, Anxiety, and Depression Symptoms (T1, T2, T3, and T4)

An abbreviated version of the Achenbach Child Behavior Checklist (CBCL) was available to assess aggression, anxiety, and depression symptoms (Achenbach, 1991). Items in this measure are rated on a three-point scale (0 = *not true* to 2 = *very true*). Mothers and fathers reported their child’s aggressive (18 items), anxiety (6 items), and depression symptoms (6 items)^3^. Aggression, anxiety, and depression symptoms were study outcomes at T3 and T4 and were covariates (to control for baseline levels of symptoms) at T1 and T2. Mothers’ and fathers’ reports were moderately to highly correlated for aggression (*r*_*T3*_ = 0.55, *r*_*T4*_ = 0.50, *ps* = < 0.001), anxiety (*r*_*T3*_ = 0.37, *r*_*T4*_ = 0.37, *ps* = < 0.001), and depression symptoms (*r*_*T3*_ = 0.36, *r*_*T4*_ = 0.39, *ps* = < 0.001). Thus, we created composite scores by averaging mother and father scores together for each outcome. Reliability was adequate in the current study (see Table 1). The CBCL has been used extensively in international research and has strong, well-documented psychometric properties (e.g., Achenbach & Rescorla, 2006).

### Procedure

We recruited participants from a range of public and private schools serving low- to high-income families by sending letters about the study home with students for their parents to read, sign, and return if they were interested in participating. We contacted interested parents via phone to schedule interviews at a location of their choosing.

We used a rigorous procedure of independent translation to ensure linguistic and conceptual equivalence across English, Spanish, and Italian versions of our measures (Erkut, 2010). Questionnaires were forward- and back-translated by graduate students, faculty, or staff members at the collaborating institutions who were fluent in English and the target language (Spanish or Italian). Translators were asked to (1) note where items did not translate well or were culturally insensitive/inappropriate, (2) identify words that elicited multiple meanings, (3) offer suggestions for improvement for identified problems, and (4) indicate reasons for suggesting changes. Site principal investigators (who were native to the country in which data was being collected and were faculty members at collaborating universities) met with the translators to review item-level discrepancies and ambiguities and modify items as needed. Our translation methods were designed to ensure measures were valid in all sites regarding linguistic equivalence as well as cultural meanings imparted by the measures (Erkut, 2010; Peña, 2007).

Before completing questionnaires, parents provided informed consent and youth provided informed assent to participant. Parent and youth participants completed questionnaires either on their own or with interviewers reading questions aloud and recording their answers. Interviewers were trained graduate students or staff members at the collaborating universities. Parent surveys took approximately 30 min to complete, and youth surveys took approximately 1½ hours to complete. We provided modest financial compensation in amounts deemed acceptable by Institution Review Boards (IRB) at each university. IRBs within each region/country approved all testing procedures and protocols (Duke University, Sapienza University of Rome, Federico II Second University of Naples, and Universidad San Buenaventura).

### Data Analytic Plan

Our aim was to examine whether HR mediated the relation of HAB to aggression, anxiety, and depression symptoms, as well as whether HAB mediated the relation of HR to aggression, anxiety, and depression symptoms. HR was assessed at only a single timepoint (T2), so the timing varied between the two types of mediation models. Specifically, models testing HR as a mediator assessed HAB at T1, HR at T2, and outcomes at T3, while models testing HAB as a mediator assessed HR at T2, HAB at T3, and outcomes at T4.

Mediation model coefficients were obtained using M*plus* 8.7 with full information maximum likelihood (FIML) estimation. FIML accounts for missing data by using all available data from each case to estimate parameters and adjust for potential bias in the estimates resulting from missing data. This approach provides unbiased estimates in the presence of data missing at random and has been identified as one of the optimal ways to handle missing data (Peugh & Enders, 2004). Mediation analyses controlled for youth baseline age (to control for developmental differences as a possible confounding variable), youth gender (to control for differences between boys and girls that may occur on our predictors and outcomes), and baseline levels of the outcome as covariates. We do not control for baseline levels of the mediator to ensure that all structural paths were parallel between our two models (see below). Indirect effects were tested using bootstrap confidence intervals (based on 5,000 resamples).

We used structural equation modeling (SEM) in our analyses to improve the precision of our estimates by modelling the measurement errors and unexplained variances (Hoyle & Smith, 1994). However, our sample size was on the lower end of what would be considered acceptable for SEM (O’Rourke & Hatcher, 2013). We therefore decided to combine scale items into parcels prior to performing our analyses to reduce the number of indicators in our models, stabilize our parameter estimates, and make our models more likely to converge (Matsunaga, 2008). This approach is particularly beneficial in multigroup analyses, where model complexity increases as additional groups are included in the comparisons. Parceling reduces the number of parameters that need to be estimated across groups, making the models more manageable, especially in the context of smaller sample sizes (Little et al., 2013).

For each latent construct (HAB, HR, aggression, anxiety, and depression), we created three domain-representative parcels by averaging conceptually similar items (Kishton & Widaman, 1994). Intentionally designed parcels are typically preferred because they provide meaning to the parcels and help reduce problematic sources of variance like correlated residuals or cross-loadings, which might otherwise confound the measurement model (Little et al., 2022). Intentional parceling commonly leads to more accurate parameter estimates and enhanced predictive validity. Parcels for HAB pertained to three different peer provocation contexts: personal property being ruined (3 items), physical discomfort (4 items), and schoolwork being wrecked (3 items). Parcels for HR pertained to three domains: sustaining/escalating hostility (3 items), remembering insults (2 items), and not forgiving insults (2 items). Parcels for aggression pertained to defiance (6 items), physical aggression (6 items), and negativity (6 items). Parcels for anxiety pertained to social doubts (2 items), perfectionism (2 items), and general apprehension (2 items). Parcels for depression pertained to isolation (2 items), low mood (2 items), and low self-worth (2 items). For measures of psychological adjustment (aggression, anxiety, and depressive symptoms), we defined the parcels the same way for both the baseline and outcome assessments. This consistency is critical for testing measurement invariance, a necessary step in multigroup analyses (Little et al., 2022). To measure model fit, we considered the χ^2^ statistic, RMSEA (Root Mean Square Error of Approximation), CFI (Comparative Fit Index), and SRMR (Standardized Root Mean Square Residual) (see Kenny, 2015). Better models are associated with higher CFI and lower χ^2^, RMSEA, and SRMR values.

We were interested in examining whether our mediation models applied similarly across our national, regional, and racial subgroups, as well as across genders. Given that the diversity within our sample is a critical source of variability in our variables of interest, we believe that comparing model fit across subgroups provides a unique and robust examination of whether meditational pathways function similarly among diverse youth. The multigroup analyses tested the two coefficients composing the mediation effect (the relation of the predictor to the mediator and the relation of the mediator to either aggression, depression symptoms, or anxiety symptoms after controlling for the predictor) as well as the direct effect of the predictor on either aggression, depression symptoms, or anxiety symptoms independent of the mediated effect. The multigroup analyses tested whether a model allowing these coefficients to be estimated independently within each subgroup fit the data significantly better than a model where these coefficients were constrained to be the same across subgroups. If constraining the coefficients to be equal across subgroups did not significantly worsen model fit, we concluded our mediation effects were consistent across subgroups. Coefficients for the covariates (i.e., baseline symptoms and age) were allowed to freely vary among groups in all models.

## Results

### Descriptive Statistics and Bivariate Correlations

Table 1 presents descriptive statistics for study variables. For all variables of interest, mean scores were below each scale’s mid-point, suggesting mild severity or frequency of HAB, HR, aggression, depression, and anxiety. Graphical and statistical examinations of normality (i.e., univariate skewness < 2.5, kurtosis < 7.0) did not suggest any substantial deviations from normality for numeric variables (see Supplemental Materials Table S2). Table 1 also presents overall reliability estimates for all composites (reliability estimates within each subsample are presented in Supplemental Materials Table S3 and reliability estimates separately by gender are presented in Supplemental Materials Table S4) (Table 2).

**Table 2:** Bivariate correlations for theoretical variables

	1	2	3	4	5	6	7	8	9	10	11	12	13	14	15	16	17
	Gender	T1 Age	T1 Agg	T1 Dep	T1 Anx	T1 HAB	T2 HR	T2 Agg	T2 Dep	T2 Anx	T3 HAB	T3 Agg	T3 Dep	T3 Anx	T4 Agg	T4 Dep	T4 Anx
1	--	− 0.01	− 0.02	0.08	0.07	**− 0.09**	***0.14***	− 0.01	**0.12**	0.00	− 0.04	0.04	***0.18***	**0.12**	0.05	***0.27***	**0.12**
2	− 0.01	--	**− 0.09**	− 0.04	***− 0.14***	0.07	0.01	**− 0.11**	**− 0.09**	**− 0.12**	− 0.06	**− 0.09**	− 0.03	− 0.11	− 0.07	0.01	− 0.05
3	− 0.02	**− 0.09**	--	***0.61***	***0.53***	***0.12***	***0.18***	***0.67***	***0.38***	***0.35***	**0.10**	***0.65***	***0.31***	***0.36***	***0.54***	***0.28***	***0.32***
4	0.08	− 0.04	***0.61***	--	***0.60***	0.07	***0.16***	***0.41***	***0.52***	***0.42***	0.04	***0.41***	***0.54***	***0.44***	***0.36***	***0.49***	***0.42***
5	0.07	***− 0.14***	***0.53***	***0.60***	--	0.07	**0.09**	***0.34***	***0.38***	***0.58***	0.02	***0.36***	***0.40***	***0.62***	***0.37***	***0.37***	***0.58***
6	**− 0.09**	0.07	***0.12***	0.07	0.07	--	***0.14***	0.06	0.04	− 0.01	***0.33***	0.01	− 0.03	**− 0.10**	0.01	− 0.07	− 0.05
7	***0.14***	0.01	***0.18***	***0.16***	**0.09**	***0.14***	--	***0.17***	**0.11**	**0.09**	***0.16***	***0.19***	***0.13***	**0.12**	***0.19***	***0.15***	**0.11**
8	− 0.01	**− 0.11**	***0.67***	***0.41***	***0.34***	0.06	***0.17***	--	***0.60***	***0.50***	0.07	***0.71***	***0.37***	***0.33***	***0.60***	***0.29***	***0.30***
9	**0.12**	**− 0.09**	***0.38***	***0.52***	***0.38***	0.04	**0.11**	***0.60***	--	***0.57***	0.06	***0.40***	***0.56***	***0.41***	***0.34***	***0.47***	***0.40***
10	0.00	**− 0.12**	***0.35***	***0.42***	***0.58***	− 0.01	**0.09**	***0.50***	***0.57***	--	− 0.02	***0.33***	***0.38***	***0.57***	***0.29***	***0.30***	***0.56***
11	− 0.04	− 0.06	**0.10**	0.04	0.02	***0.33***	***0.16***	0.07	0.06	− 0.02	--	**0.11**	0.02	0.01	0.05	− 0.02	**− 0.09**
12	0.04	**− 0.09**	***0.65***	***0.41***	***0.36***	0.01	***0.19***	***0.71***	***0.40***	***0.33***	**0.11**	--	***0.51***	***0.48***	***0.72***	***0.38***	***0.35***
13	***0.18***	− 0.03	***0.31***	***0.54***	***0.40***	− 0.03	***0.13***	***0.37***	***0.56***	***0.38***	0.02	***0.51***	--	***0.59***	***0.44***	***0.65***	***0.49***
14	**0.12**	− 0.11	***0.36***	***0.44***	***0.62***	**− 0.10**	**0.12**	***0.33***	***0.41***	***0.57***	0.01	***0.48***	***0.59***	--	***0.44***	***0.45***	***0.70***
15	0.05	− 0.07	***0.54***	***0.36***	***0.37***	0.01	***0.19***	***0.60***	***0.34***	***0.29***	0.05	***0.72***	***0.44***	***0.44***	--	***0.53***	***0.49***
16	***0.27***	0.01	***0.28***	***0.49***	***0.37***	− 0.07	***0.15***	***0.29***	***0.47***	***0.30***	− 0.02	***0.38***	***0.65***	***0.45***	***0.53***	--	***0.60***
17	**0.12**	− 0.05	***0.32***	***0.42***	***0.58***	− 0.05	**0.11**	***0.30***	***0.40***	***0.56***	**− 0.09**	***0.35***	***0.49***	***0.70***	***0.49***	***0.60***	--

*Note* Bivariate correlations above the diagonal are the same as those below the diagonal. Gender is coded as 1 = female and 0 = male; HAB = hostile attribution bias; HR = hostile rumination; Agg = aggression, Dep = depression symptoms, Anx = anxiety symptoms; Reliability estimates and correlations separately by subgroup and gender are available from the first author. Bold font indicates *p* *≤*.05 and bold italicized font indicates *p* *≤*.005

### Missing Data Analyses

We had relatively low missingness on study variables. Missingness on focal variables (HAB, HR, depression, anxiety, aggression) ranged from 0 to 8.7% with a median of 4.1%. Out of the 566 cases in our data set, 473 (84%) had complete data. Twenty-four participants (4.2%) were only missing values at Time 4, and twenty-one participants (3.7%) only had values at Time 1. No other pattern of missingness represented more than 2% of the cases. Our analyses do not provide us with reason to suspect the presence of non-random missingness in our data.

### HAB Through HR to Outcomes

**Fig. 1:**
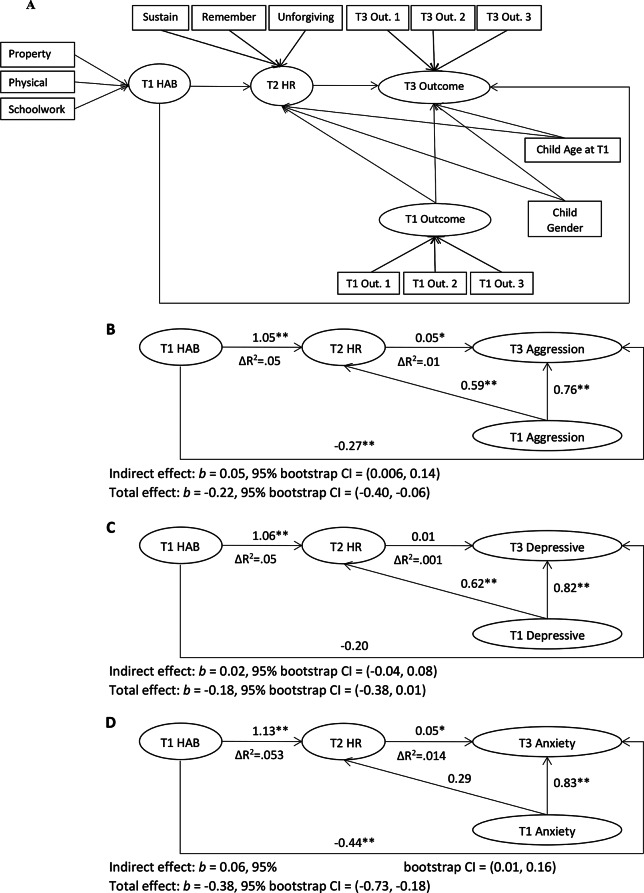
(**A**) Structural equation model used for all mediational pathways from hostile attribution bias (HAB) through hostile rumination (HR) to outcomes. Covariates (child age at time 1 and child gender) and parcels are included in all analyses but are not depicted in B-D. (**B**) Aggression outcome model. (**C**) Depressive symptoms outcome model. (**D**) Anxiety symptoms outcome model. Property = personal property ruined, physical = physical discomfort, schoolwork = schoolwork wrecked; sustain = sustaining/escalating hostility, remember = remembering insults, unforgiving = unforgiving of insults. Parcels for outcomes were as follows: T3 & T1 aggression 1 = defiance, 2 = physical aggression, 3 = negativity; T3 & T1 anxiety 1 = social doubts, 2 = perfectionism, 3 = general apprehension; T3 & T1 depression 1 = isolation, 2 = low mood, 3 = low self-worth. Unstandardized coefficients are displayed. *p <.05, **p <.01

Figure 1A depicts the overall analytic model used to test the ability of HR to mediate the relations of HAB to aggression, anxiety, and depression symptoms. All analyses controlled for baseline levels of the outcome, child age at baseline, and child gender. The model for aggression had tolerable fit to the data, χ^2^(64) = 262.35, *p* <.001, CFI = 0.92, RMSEA = 0.07, 90% CI = (0.06, 0.08), SRMR = 0.04. HR significantly mediated the relation of HAB to parent-reported aggression. Higher levels of HAB at T1 were associated with higher levels of HR at T2, which were in turn associated with more parent-reported aggression at T3 (see Fig. 1B).

The model for depression had acceptable fit to the data, χ^2^(64) = 148.82, *p* <.001, CFI = 0.93, RMSEA = 0.05, 90% CI = (0.04, 0.06), SRMR = 0.04. HR did not significantly mediate the relation of HAB to parent-reported depression symptoms. Although HAB at T1 predicted HR at T2, HR did not predict depressive symptoms at T3 (see Fig. 1C).

The model for anxiety had tolerable fit to the data, χ^2^(64) = 202.77, *p* <.001, CFI = 0.92, RMSEA = 0.06, 90% CI = (0.05, 0.07), SRMR = 0.04. HR significantly mediated the relation of HAB to parent-reported anxiety symptoms. Higher levels of HAB at T1 were associated with higher levels of HR at T2, which were associated with more anxiety symptoms at T3 (see Fig. 1D).

### HR Through HAB to Outcomes

**Fig. 2:**
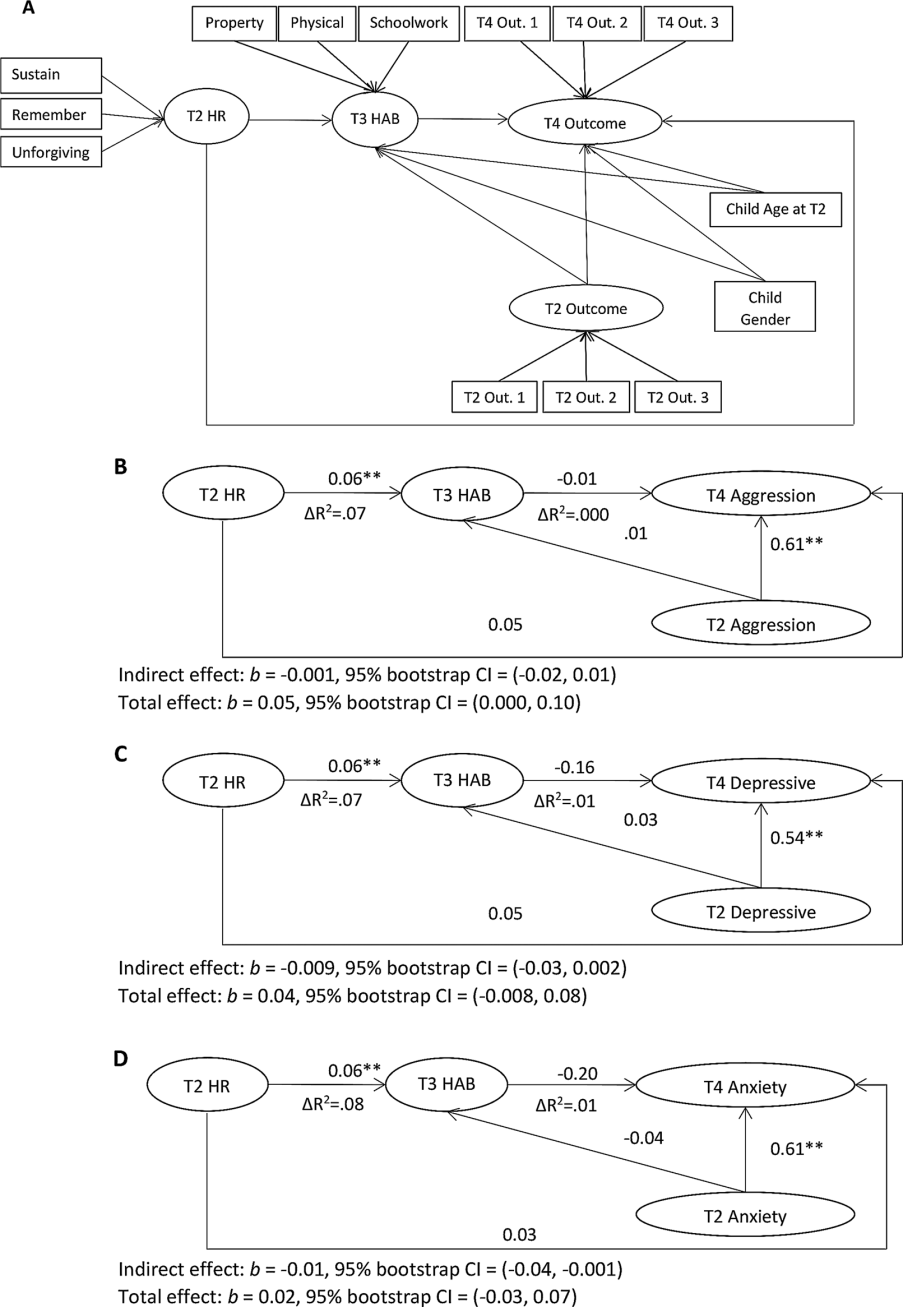
(**A**) Structural equation model used for all mediational pathways from hostile rumination (HR) through hostile attribution bias (HAB) to outcomes. Covariates (child age at time 2 and child gender) and parcels are included in all analyses but are not depicted in B-D. (**B**) Aggression outcome model. (**C**) Depressive symptoms outcome model. (**D**) Anxiety symptoms outcome model. Property = personal property ruined, physical = physical discomfort, schoolwork = schoolwork wrecked; sustain = sustaining/escalating hostility, remember = remembering insults, unforgiving = unforgiving of insults. Parcels for outcomes were as follows: T4 & T2 aggression 1 = defiance, 2 = physical aggression, 3 = negativity; T4 & T2 anxiety 1 = social doubts, 2 = perfectionism, 3 = general apprehension; T4 & T2 depression 1 = isolation, 2 = low mood, 3 = low self-worth. Unstandardized coefficients are displayed. *p <.05, **p <.01

Figure 2A depicts the overall analytic model used to test the ability of HAB to mediate the relations of HR to aggression, anxiety, and depression symptoms. The model for aggression had tolerable fit to the data, χ^2^(64) = 283.09, *p* <.001, CFI = 0.90, RMSEA = 0.07, 90% CI = (0.07, 0.08), SRMR = 0.04. HAB did not significantly mediate the relation of HR to parent-reported aggression. Unstandardized coefficients for individual pathways in this model are depicted in Fig. 2B. The model for depression had an acceptable fit to the data, χ^2^(64) = 181.64, *p* <.001, CFI = 0.90, RMSEA = 0.05, 90% CI = (0.05, 0.06), SRMR = 0.04. HAB did not significantly mediate the relation of HR to parent-reported depression symptoms (see Fig. 2C). The model for anxiety had acceptable fit to the data, χ^2^(64) = 177.71, *p* <.001, CFI = 0.93, RMSEA = 0.05, 90% CI = (0.04, 0.06), SRMR = 0.04. HAB significantly mediated the relation of HR to parent-reported anxiety symptoms; however, the direction of this indirect effect was negative (see Fig. 2D^4^).

### Parcel Allocation Variability

It can be useful to assess parcel allocation variability, or the extent to which the analytic results vary depending on how the parceling was performed (Sterba & Right, 2016). We therefore reran our HAB through HR to psychological maladjustment models and our HR through HAB to psychological maladjustment models 100 times each, where we randomly assigned the HAB, HR, and psychological maladjustment items to each parcel. The variability in coefficients across these models is presented in Supplemental Material Table S5, whereas the percent of the random parcel models identifying significant mediation effects is presented in Table 3. Consistent with our primary analyses, a clear majority of the HAB through HR to aggression and the HAB through HR to anxiety random parcel models showed significant mediation, whereas a clear majority of the HAB through HR to depression symptoms models did not. Although we identified a significant HR through HAB to anxiety symptoms effect in our primary analysis, none of the HR through HAB to anxiety symptoms random parcel models showed significant mediation, suggesting that we should be cautious about drawing conclusions from this result. Consistent with our primary analyses, none of the HR through HAB to aggression random parcel models or the HR through HAB to depression symptoms random parcel models showed significant mediation.

**Table 3:** Parcel allocation variability in indirect effects

	Percent of models where the mediation effect was significant		
	Aggression	Depression	Anxiety
HAB to HR to Outcome	98%	8%	95%
HR to HAB to Outcome	0%	0%	0%

*Note* Significance was identified when the 95% bootstrap confidence interval around the indirect effect did not include 0

### Multigroup Analyses

We used multigroup analyses to determine whether the focal coefficients in our models varied significantly across our six samples separately for each outcome. These analyses were performed by comparing the fit of a model constraining the mediation coefficients and the direct effect to be equal across groups to a model allowing these coefficients to vary across groups. We did not find any evidence that these coefficients significantly varied across samples for the HAB through HR to outcomes models (all *ps* > 0.58) or for the HR through HAB to outcomes models (all *ps* > 0.22). An examination of changes in alternate fit indices also did not show evidence of substantial group differences (HAB through HR to outcomes: all ΔRMSEA ≤ 0.000, all ΔCFI ≤ 0.000, all ΔSRMR ≤ 0.001; HR through HAB to outcomes: all ΔRMSEA ≤ 0.000, all ΔCFI ≤ 0.003, all ΔSRMR ≤ 0.002; see Chen, 2007). Details of these analyses are provided in Supplemental Table S6.

We also used multigroup analyses to determine whether the mediation coefficients in our models significantly varied across male and female participants. Using the same procedures as for comparing samples, we did not find any evidence that the mediation coefficients and direct effects significantly varied across gender (HAB through HR to outcomes: all *ps* > 0.09, all ΔRMSEA ≤ 0.001, all ΔCFI ≤ 0.003, all ΔSRMR ≤ 0.003; HR through HAB to outcomes: all *ps* > 0.30, all ΔRMSEA ≤ 0.000, all ΔCFI ≤ 0.000, all ΔSRMR ≤ 0.002). Details of these analyses are provided in Supplemental Table S7.

## Discussion

Given growing concerns about the mental health of youth around the globe (United Nations, 2020), it is imperative to identify mechanisms explaining pathways to psychological maladjustment among culturally and racially diverse youth within a developmental period highly amenable to change. Aligned with integrative models of aggression (Dodge, 2006; Wilkowski & Robinson, 2008), the current study investigated HR as a cognitive mechanism explaining how HABs relate to aggression, depression, and anxiety symptoms over time. We also tested competing mediational pathways involving these cognitive mechanisms. Our samples of early adolescents from three countries and six subgroups allowed us to test the generalizability of these pathways to psychological maladjustment.

HR significantly mediated the relation of HAB to aggression. Youths making more hostile attributions at age 11 engaged in more HR at age 12 and consequently engaged in more aggression at age 13 (controlling for aggression levels at age 11, child age, and child gender). Our results are consistent with theory and prior empirical findings aligned with integrated models of aggression (Dodge, 2006; Quan et al., 2019; Wilkowski & Robinson, 2008). Evidence from the current study expands these models by providing scientific evidence that they apply to early adolescents in addition to adults.

HR also mediated the relation of HAB to anxiety symptoms. Youths making more hostile attributions at age 11 engaged in more HR at age 12 and consequently experienced more anxiety symptoms at age 13 (controlling for anxiety levels at age 11, child age, and child gender). This finding suggests the Integrated Cognitive Model of Aggression (Wilkowski & Robinson, 2008) could be extended to more psychological adjustment outcomes than originally theorized, particularly among youth.

HR did not significantly mediate the relation of HAB to depressive symptoms. Depression has a later age of onset relative to anxiety and aggression-related disorders (i.e., adolescence for anxiety, conduct disorder, and oppositional defiant disorder; Kessler et al., 2005). Furthermore, Hankin and colleagues (2015) found that depression symptoms increase substantially between 14 and 17 years and are especially higher among post-pubertal youth. Youths in our samples were between the ages of 10 to 14 years, and only 18% of our sample was post-pubertal at age 13 (based on the pubertal development scale by Petersen et al., 1988). Thus, our sample may have been too young for us to detect changes in depressive symptoms across time. Future research should examine whether HAB and HR serve as pathways to depressive symptoms among older adolescents and young adults. Equally plausible is that the Integrated Cognitive Model of Aggression (Wilkowski & Robinson, 2008) might extend only to specific types of maladjustment. Further investigations of how broadly this model applies to different forms of psychological maladjustment among youth are warranted.

Findings from our study are consistent with prior evidence indicating that HR predicts aggression among diverse youth (Caprara et al., 2007; Harmon et al., 2019). Multigroup analyses indicated mediation and direct effects were similar across our six national/regional/racial subgroups and gender, demonstrating that HR is one mechanism predicting the relation of HAB to aggression across nationally, regionally, and racially diverse boys and girls. Consequently, the applicability of the Integrated Cognitive Model of Aggression can be expanded to include socio-culturally diverse youth. Beyond aggression, our findings offer evidence that HR might also be a mechanism explaining the relation of HAB to anxiety symptoms. Collectively, these findings suggest that HR might be a transdiagnostic mediator of pathways from HAB to multiple forms of psychological maladjustment for youth from multiple socio-cultural backgrounds. Translated into practice, these findings could improve prevention and treatment efforts, potentially enhancing the wellbeing of diverse youth from around the world.

We also tested the reverse hypothesis that HAB mediates the relation of HR to later psychological symptoms (i.e., aggression, depressive symptoms, and anxiety symptoms). HAB was a significant mediator of the HR to anxiety symptoms relation, although this indirect effect was negative, opposite in direction from the indirect effect from HAB to HR to anxiety. Additionally, the HAB to HR to anxiety mediation was not replicated in our random parcel models, suggesting caution when interpreting this effect. Neither the indirect effect from HR to HAB to aggression nor the indirect effect from HR to HAB to depressive symptoms was significant. Given we found no evidence that HR predicts HAB in a way that leads to increases in aggression or anxiety, the reverse hypothesis does not offer a plausible alternative explanation for our observed ability of HR to mediate the relation of HAB to aggression and anxiety.

We found negative direct effects of HAB on aggression and anxiety symptoms after controlling for the indirect effect through HR. Although the negative direct effect for anxiety was consistent with the bivariate correlation of HAB with anxiety, the negative direct effect for aggression was inconsistent with the bivariate correlation of HAB with aggression, suggesting a suppression effect (Woolley, 1997). These negative direct effects operate in opposition to the positive indirect effects, leading to total effects that were negative. Given that coefficients in regression and path models represent the partial relations of each predictor to the outcome (Cohen et al., 2003), the negative direct effects suggest that HAB affects aggression and anxiety through multiple independent pathways, some of which increase the presence of these symptoms (such as the pathway through HR), whereas others decrease the presence of these symptoms (which are unidentified in our model and incorporated in the direct effect). The identification of mechanisms behind the negative pathways could be a valuable direction for future research.

Our findings suggest integrated interventions targeting both HAB and HR could provide some benefits over intervening on either construct alone. Evidence from interventions targeting attribution biases (like HAB) demonstrate wide variability in their effectiveness at reducing aggression and anxiety symptoms (Dodge et al., 2013; Hiemstra et al., 2019; Martinelli et al., 2022). Although hostile attributions are modifiable, they are difficult to change because they develop out of years of learning experiences that begin early in life (Dodge, 2006). Given the differential effectiveness of interventions across different participants, having a multi-pronged intervention could more effectively prevent the potential negative impacts of HAB. Specifically, for participants with less malleable HABs, the intervention components focused on HR could interrupt the positive pathway leading from HAB to psychological maladjustment through HR. Assigning intent to actors in social situations occurs in the moment of a social encounter. Our findings indicate that it is also important to examine how youth process social encounters after they occur, such as by ruminating, as an outcome of in-the-moment processing, such as attribution biases. To more fully alter pathways leading to negative psychological outcomes, interventions could address both negative attributions made in the moment and the consequent negative rumination that occurs after the social encounter is over.

The Integrated Cognitive Model of Aggression (Wilkowski & Robinson, 2008) provides theoretical guidance on what mechanisms might decrease both negative rumination and HABs. Both re-appraising HABs and using self-distraction to terminate hostile ruminative thinking require effortful control (Wilkowski & Robinson, 2008). Effortful control involves conscious, intentional processing and is used both to shift attention as well as to inhibit inappropriate dominant behavioral responses in favor of less dominant but more appropriate reactions (Alessandri et al., 2022). By intentionally disengaging from automatic processes and recruiting controlled processes via effortful control, youth could more accurately interpret ambiguous negative social interactions as benign and redirect their attention away from ruminating on hostile, retribution-focused thoughts (Wilkowski & Robinson, 2008). Examining mechanisms, such as effortful control, that might reduce both negative attributions and rumination could be a valuable direction for future research with important implications for psychological interventions with youth.

### Limitations and Future Directions

An important limitation of our study was that HR was only assessed at a single point in time. When performing a longitudinal mediation analysis, best practices suggest that the model equations control for earlier assessments of the mediator and the outcome (Cole & Maxwell, 2003). Although our mediation models control for baseline assessments of the outcomes, we lacked the ability to control for baseline values of HR when it was acting as a mediator. Future investigators could provide more compelling causal evidence for the contributions of HR and HAB to psychological maladjustment if they examine the presence of mediation in a cross-lagged panel mediation model involving HR, HAB, and the outcomes, where both mediation models could be examined using the most appropriate controls.

Our study used self-report measures of HAB and HR that only capture youths’ explicit awareness of these cognitive processes. There are aspects of HAB and HR that occur beyond one’s explicit awareness and are not captured by self-report assessments (Prinstein et al., 2005). It is important for future studies to supplement self-report measures with assessments capturing implicit automatic processes such as selective attention to hostile information (Pollak & Tolley-Schell, 2003) or accessibility of hostile thoughts (Lindsay & Anderson, 2000). Another limitation to note is that although our measures of anxiety and depressive symptoms were theoretically derived from the CBCL, they were not validated CBCL scale scores. Finally, the reliability of HAB in our study was low, so we must be cautious about interpreting non-significant relations. Future research should focus on improving the assessment of HAB to enable clearer and more accurate interpretations.

Future studies of HAB employing community samples of youth might examine general beliefs not tethered to negative perceptions of others. One such example can be found in Yeager et al. (2013), who demonstrated that building beliefs that personality traits are flexible and changeable can reduce the likelihood youth ascribe negative intentions to others’ ambiguous behaviors. Reducing psychological maladjustment by targeting cognitive vulnerabilities such as HAB and HR might also be more effective for community samples of youth (e.g., Vassilopoulos et al., 2014) than youth with clinical levels of maladjustment (Hiemstra et al., 2019), indicating the importance of prevention. Corroborating our findings among youth with clinically elevated aggression, anxiety, and depression symptoms would be particularly informative for developing evidence-based treatments.

Additional contextual, cognitive, and emotional factors exist downstream from HAB and HR that we did not examine. For example, negative early life experiences with parents (e.g., chronic physical abuse) and peers (e.g., chronic social rejection) predict hostile and defensive patterns of processing social information as well as psychological maladjustment (Dodge et al., 2022). Other cognitive and emotional factors likely predict HAB and HR, such as irritability (Leigh et al., 2020). Future studies could examine whether irritability mediates the relation of HR to psychological maladjustment and assess the utility of a serial mediation model suggesting that HAB influences HR, which leads to irritability, which finally leads to psychological maladjustment. Existing evidence supports such a model. For example, irritability predicts internalizing and externalizing symptoms in adolescence (Sorcher et al., 2022), is associated with biases toward anger (Salum et al., 2017), and is associated with HR (Caprara et al., 2007).

## Conclusion

Early adolescence is an important developmental window for interventions targeting putative cognitive vulnerabilities associated with psychological maladjustment (Dodge, 2006). Our longitudinal findings from nationally, regionally, and racially/ethnically diverse youth indicate HR is an important transdiagnostic mechanism through which HAB relates to aggression and anxiety symptoms. Our findings offer several avenues for future research and suggest possible strategies to foster healthy psychological adaptation for diverse youth across multiple cultures. Although our results do not prove causality, they suggest that the effectiveness of interventions targeting psychological adaptation might be enhanced by strategies building youths’ skills in accurately assigning intent to individuals in ambiguous encounters and in redirecting their perseverative thinking to non-hostile content.

## Electronic Supplementary Material

Below is the link to the electronic supplementary material.

Supplementary Material 1

## Data Availability

De-identified data, materials, and code from analyses are available from the first author.

## Footnotes

1 We acknowledge that HAB and negative interpretation bias refer to different but theoretically related cognitive processing biases. We therefore use the term HAB when the interpretation bias is specific to hostility and the term negative interpretation bias to refer to more general negative interpretations that may or may not include hostility among other interpretations such as social rejection or scrutiny.

2 Comparable racial/ethnic data were not available in Italy or Colombia.

3 Authorized CBCL users can request the item numbers for items comprising our anxiety and depression subscale scores from the first author.

4 We also replicated our proposed mediation models using path analysis where each latent construct was replaced with a mean composite and obtained conceptually similar results. Specifically, HR significantly mediated the relations of HAB to aggression and anxiety, whereas HAB did not significantly mediate the relations of HR to any of our outcomes. Focal coefficients and indirect effect confidence intervals from these models are presented in Supplemental Materials Figure S1.
