# Supplementary material for: Rumination Mediates the Relation of Hostile Attribution to Psychological Maladjustment Among Adolescents from Three Countries
Source: Res Child Adolesc Psychopathol. 2025 Jan 31;53(6):861–76. doi: 10.1007/s10802-025-01288-z (PMC12137534; doi:10.1007/s10802-025-01288-z)
Supplement: Supplementary file 1 — Supplementary Material 1 [file 10802_2025_1288_MOESM1_ESM.docx]

# Supplemental Materials

# Table S1.

*Socio-demographic information separately by group.*

|  | **Colombia** | **Italy** | | **United States** | | |
| --- | --- | --- | --- | --- | --- | --- |
|  |  | **Rome** | **Naples** | **White** | **Black** | **Latinx** |
| Marital status (%) | | | | | | |
| Married | 69.4 | 75.5 | 83 | 81.3 | 37.2 | 55.7 |
| Remarried | 0 | 0 | 2.2 | 2 | 1.2 | 2.9 |
| Divorced | 0 | 1.1 | 1.1 | 7.2 | 9.3 | 7.1 |
| Separated | 3.4 | 11.1 | 6.8 | 2.1 | 7 | 7.1 |
| Widowed | 0 | 2 | 2.3 | 2.2 | 1.2 | 0 |
| Cohabiting | 22.7 | 3.1 | 2.3 | 2 | 7 | 18.6 |
| Never married | 4.5 | 7.2 | 2.3 | 3.2 | 37.1 | 8.6 |
| Education level [Mean(SD)] |  |  |  |  |  |  |
| Mothers | 10.64(5.21) | 13.52(4.00) | 10.46(4.44) | 16.69(3.07) | 13.69(2.25) | 10.11(4.13) |
| Fathers | 9.67(4.91) | 13.80(3.69) | 11.39(4.65) | 16.86(3.22) | 13.71(2.59) | 9.72(4.47) |
| Family income in Euros/Dollars; Colombian Pesos (%) | | | | | | |
| Up to 5,000; Up to 5,000,000 | 22.7 | 5.1 | 18.0 | 1.1 | 8.2 | 6.1 |
| 5,000 – 10,000; 5,000,000 – 10,000,000 | 33.0 | 4.1 | 18.0 | 1.1 | 5.9 | 7.6 |
| 11,000 – 15,000; 11,000,000 – 16,000,000 | 13.6 | 8.2 | 16.9 | 1.1 | 7.1 | 21.2 |
| 16,000 – 29,000; 17,000,000 – 24,000,000 | 4.5 | 29.6 | 23.6 | 6.3 | 29.4 | 40.9 |
| 30,000 – 40,000; 25,000,000 – 30,000,000 | 3.4 | 21.4 | 7.9 | 6.3 | 17.6 | 12.1 |
| 41,000 – 50,000; 31,000,000 – 40,000,000 | 3.4 | 8.2 | 5.6 | 7.4 | 8.2 | 3.0 |
| 51,000 – 60,000; 41,000,000 – 50,000,000 | 2.3 | 6.1 | 5.6 | 10.5 | 9.4 | 3.0 |
| 61,000 – 70,000; 51,000,000 – 60,000,000 | 4.5 | 7.1 | 2.2 | 12.6 | 1.2 | 1.5 |
| 71,000 – 80,000; 61,000,000 – 70,000,000 | 5.7 | 4.1 | 1.1 | 53.7 | 2.4 | 4.5 |
| Above 81,000; Above 71,000,000 | 6.8 | 6.1 | 1.1 | 1.1 | 10.6 | 6.1 |
| Work Status: Mothers | | | | | | |
| % working | 50.0 | 78.8 | 42.1 | 75.5 | 65.6 | 63.8 |
| % of those working who work full time | 45.9 | 61.5 | 52.5 | 56.8 | 58.8 | 40.6 |
| Work Status: Fathers | | | | | | |
| % working | 92.9 | 92.6 | 90.2 | 95.5 | 83.0 | 87.7 |
| % of those working who work full time | 81.7 | 98.9 | 90.5 | 94.3 | 73.5 | 65.1 |

**Table S2.**

*Descriptive statistics—Skew and kurtosis.*

|  | Skew (SE) | Kurtosis (SE) |
| --- | --- | --- |
| Gender | 0.00 (.10) | -2.01 (.21) |
| T1 Age | -0.06 (.10) | -0.07 (.21) |
| T1 Hostile attribution bias | -0.16 (0.10) | -0.44 (0.21) |
| T3 Hostile attribution bias | 0.01 (0.11) | -0.33 (0.21) |
| T2 Hostile rumination | -0.10 (0.11) | -0.26 (0.21) |
| T1 Aggression | 1.71 (.10) | 4.72 (.21) |
| T1 Depression | 1.95 (.10) | 5.86 (.21) |
| T1 Anxiety | 0.60 (.10) | 0.21 (.21) |
| T2 Aggression | 1.49 (.11) | 2.44 (.21) |
| T2 Depression | 1.91 (.11) | 4.15 (.21) |
| T2 Anxiety | 0.72 (.11) | -0.13 (.21) |
| T3 Aggression | 1.49 (.11) | 3.71 (.21) |
| T3 Depression | 1.93 (.11) | 6.05 (.21) |
| T3 Anxiety | 0.58 (.11) | -0.18 (.21) |
| T4 Aggression | 1.62 (.11) | 3.87 (.22) |
| T4 Depression | 2.09 (.11) | 5.98 (.22) |
| T4 Anxiety | 0.76 (.11) | 0.32 (.22) |

**Table S3.**

*Reliability estimates within each subsample*.

|  | **Cronbach’s α (McDonald’s ω)** | | | | | |
| --- | --- | --- | --- | --- | --- | --- |
|  | **Naples** | **Rome** | **U.S. Black** | **U. S. White** | **U.S. Latinx** | **Colombia** |
| HAB time 1 | .54 (.53) | .56 (.57) | .62 (.59) | .67 (.68) | .64 (.66) | .72 (.72) |
| HAB time 3 | .40 (.43) | .54 (.53) | .52 (.50) | .64 (.65) | .64 (.65) | .63 (.64) |
| HR time 2 | .58 (.57) | .65 (.65) | .79 (.79) | .76 (.76) | .79 (.80) | .65 (.65) |
| Aggression time 1 | .84 (.85) | .86 (.86) | .90 (.91) | .92 (.93) | .89 (.90) | .86 (.87) |
| Aggression time 2 | .82 (.82) | .78 (.80) | .93 (.93) | .89 (.89) | .85 (.85) | .89 (.90) |
| Aggression time 3 | .83 (.83) | .84 (.84) | .91 (.91) | .87 (.88) | .86 (.86) | .91 (.91) |
| Aggression time 4 | .84 (.84) | .87 (.88) | .93 (.93) | .87 (.87) | .83 (.82) | .90 (.91) |
| Depression time 1 | .80 (.81) | .67 (.67) | .68 (.70) | .80 (.81) | .72 (.72) | .58 (.63) |
| Depression time 2 | .75 (.75) | .60 (.64) | .69 (.72) | .74 (.75) | .74 (.77) | .57 (.66) |
| Depression time 3 | .79 (.80) | .68 (.68) | .78 (.78) | .78 (.78) | .74 (.73) | .53 (.61) |
| Depression time 4 | .72 (.74) | .85 (.86) | .80 (.80) | .84 (.85) | .66 (.64) | .76 (.80) |
| Anxiety time 1 | .72 (.74) | .74 (.75) | .64 (.69) | .84 (.84) | .72 (.73) | .71 (.72) |
| Anxiety time 2 | .74 (.74) | .69 (.70) | .67 (.66) | .81 (.81) | .58 (.60) | .73 (.72) |
| Anxiety time 3 | .66 (.66) | .73 (.73) | .75 (.77) | .83 (.84) | .72 (.73) | .72 (.72) |
| Anxiety time 4 | .72 (.73) | .78 (.79) | .82 (.83) | .86 (.87) | .52 (.61) | .68 (.68) |

**Table S4.**

*Reliability estimates by gender*.

|  | **Cronbach’s α (McDonald’s ω)** | |
| --- | --- | --- |
|  | **Female** | **Male** |
| HAB time 1 | .64 (.64) | .61 (.62) |
| HAB time 3 | .61 (.62) | .53 (.54) |
| HR time 2 | .70 (.70) | .71 (.71) |
| Aggression time 1 | .88 (.88) | .88 (.89) |
| Aggression time 2 | .87 (.87) | .88 (.88) |
| Aggression time 3 | .87 (.88) | .87 (.88) |
| Aggression time 4 | .89 (.90) | .87 (.88) |
| Depression time 1 | .75 (.76) | .71 (.71) |
| Depression time 2 | .73 (.74) | .65 (.66) |
| Depression time 3 | .78 (.78) | .62 (.63) |
| Depression time 4 | .82 (.82) | .62 (.63) |
| Anxiety time 1 | .80 (.80) | .75 (.76) |
| Anxiety time 2 | .77 (.77) | .74 (.74) |
| Anxiety time 3 | .77 (.77) | .76 (.76) |
| Anxiety time 4 | .80 (.70) | .77 (.78) |

**Table S5.**

*Parcel allocation variability in coefficients.*

| Model | Coefficient | Median *b* | Range of *b* | Median SE | Range of SE | Percent Significant |
| --- | --- | --- | --- | --- | --- | --- |
| HAB to HR to Aggression | IV to Mediator | .761 | (.473, 1.060) | .274 | (.181, .390) | 100% |
|  | Mediator to DV | .128 | (.063, .223) | .050 | (.031, .087) | 98% |
| HAB to HR to Depression | IV to Mediator | .758 | (.429, 1.069) | .272 | (.170, .392) | 100% |
|  | Mediator to DV | .020 | (.005, .049) | .016 | (.009, .027) | 91% |
| HAB to HR to Anxiety | IV to Mediator | .810 | (.450, 1.088) | .279 | (.170, .388) | 100% |
|  | Mediator to DV | .058 | (.032, .102) | .024 | (.015, .039) | 1% |
| HR to HAB to Aggression | IV to Mediator | .056 | (.034, .111) | .021 | (.013, .034) | 100% |
|  | Mediator to DV | .100 | (-040, .225) | .235 | (.113, .381) | 0% |
| HR to HAB to Depression | IV to Mediator | .056 | (.032, .109) | .020 | (.013, .033) | 100% |
|  | Mediator to DV | -.049 | (-.094, -.007) | .088 | (.038, .154) | 0% |
| HR to HAB to Anxiety | IV to Mediator | .059 | (.033, .117) | .021 | (.013, .034) | 100% |
|  | Mediator to DV | -.057 | (-.185, .025) | .123 | (.055, .211) | 0% |

*Note*. Confidence intervals are based on the 5^th^ and 95^th^ percentiles in the distribution of estimates obtained across the 100 analyses using different random parcels. Similarly, the percent significant is the percent of the 100 analyses that identified the corresponding coefficient as significant (p < .05).

**Table S6.**

# *Multigroup analysis mediation coefficients for sample.*

|  |  | IV to Mediator (a) | | Mediator to DV (b) | | IV to DV (c’) | |
| --- | --- | --- | --- | --- | --- | --- | --- |
| Model | Group | b | 95% CI | b | 95% CI | b | 95% CI |
| HAB to HR to Aggression | Fixed across samples | 0.894 | (0.102, 1.686) | 0.031 | (0.011, 0.051) | -0.292 | (-0.463, -0.121) |
| Fixed vs free test: | Naples | 0.448 | (-1.338, 2.234) | 0.047 | (-0.022, 0.116) | 0.0270 | (-0.400, 0.454) |
| χ^2^[15]=10.865, *p*=0.762 | Rome | 0.407 | (-1.776, 2.59) | 0.054 | (-0.013, 0.121) | -0.488 | (-1.145, 0.169) |
| ΔRMSEA=-.002, | U.S. Black | 0.850 | (-1.141, 2.841) | 0.011 | (-0.018, 0.040) | -0.307 | (-0.730, 0.116) |
| ΔCFI=-.002, | U.S. White | 1.757 | (0.144, 3.370) | 0.038 | (0.005, 0.071) | -0.431 | (-0.731, -0.131) |
| ΔSRMR=.001 | U.S. Latinx | -0.882 | (-3.191, 1.427) | 0.036 | (0.005, 0.067) | -0.247 | (-0.670, 0.176) |
|  | Colombia | 1.254 | (-0.259, 2.767) | 0.009 | (-0.114, 0.132) | -0.197 | (-0.656, 0.262) |
| HAB to HR to Depression | Fixed across samples | 0.920 | (0.128, 1.712) | 0.021 | (-0.001, 0.043) | -0.210 | (-0.412, -0.008) |
| Fixed vs free test: | Naples | 0.480 | (-1.253, 2.213) | 0.047 | (-0.039, 0.133) | -0.073 | (-0.614, 0.468) |
| χ^2^[15]=9.177, *p*=0.868, | Rome | 0.487 | (-1.694, 2.668) | 0.035 | (-0.016, 0.086) | -0.378 | (-1.111, 0.355) |
| ΔRMSEA=-.002, | U.S. Black | 0.669 | (-1.213, 2.551) | -0.009 | (-0.036, 0.018) | -0.274 | (-0.690, 0.142) |
| ΔCFI=-.003, | U.S. White | 1.637 | (0.010, 3.264) | 0.021 | (-0.020, 0.062) | -0.254 | (-0.728, 0.220) |
| ΔSRMR=-.001 | U.S. Latinx | -0.513 | (-2.851, 1.825) | 0.032 | (-0.005, 0.069) | -0.064 | (-0.607, 0.479) |
|  | Colombia | 1.289 | (-0.244, 2.822) | 0.048 | (-0.044, 0.140) | -0.273 | (-0.696, 0.150) |
| HAB to HR to Anxiety | Fixed across samples | 0.825 | (0.031, 1.619) | 0.026 | (0.006, 0.046) | -0.368 | (-0.576, -0.160) |
| Fixed vs free test: | Naples | 0.436 | (-1.336, 2.208) | 0.073 | (-0.001, 0.147) | -0.188 | (-0.670, 0.294) |
| χ^2^[15]=13.276, *p*=0.581, | Rome | -0.039 | (-2.45, 2.372) | 0.038 | (-0.011, 0.087) | -0.995 | (-1.938, -0.052) |
| ΔRMSEA=-.001, | U.S. Black | 0.862 | (-1.025, 2.749) | 0.006 | (-0.019, 0.031) | -0.446 | (-0.879, -0.013) |
| ΔCFI=-.001, | U.S. White | 1.552 | (-0.063, 3.167) | 0.023 | (-0.010, 0.056) | -0.436 | (-0.877, 0.005) |
| ΔSRMR=.000 | U.S. Latinx | -0.400 | (-2.703, 1.903) | 0.026 | (-0.005, 0.057) | -0.371 | (-0.949, 0.207) |
|  | Colombia | 1.272 | (-0.286, 2.83) | 0.037 | (-0.057, 0.131) | -0.204 | (-0.604, 0.196) |
| HR to HAB to Aggression | Fixed across samples | 0.023 | (0.003, 0.043) | -0.016 | (-0.218, 0.186) | 0.008 | (-0.012, 0.028) |
| Fixed vs free test: | Naples | 0.007 | (-0.040, 0.054) | -0.059 | (-1.110, 0.992) | -0.032 | (-0.106, 0.042) |
| χ^2^[15]=16.498, *p*=0.350, | Rome | 0.034 | (-0.011, 0.079) | -0.389 | (-0.957, 0.179) | 0.037 | (-0.022, 0.096) |
| ΔRMSEA=-.002, | U.S. Black | 0.010 | (-0.014, 0.034) | 0.165 | (-0.474, 0.804) | 0.017 | (-0.016, 0.050) |
| ΔCFI=.001, | U.S. White | 0.047 | (0.012, 0.082) | -0.004 | (-0.457, 0.449) | -0.003 | (-0.042, 0.036) |
| ΔSRMR=.001 | U.S. Latinx | -0.003 | (-0.030, 0.024) | 0.059 | (-0.304, 0.422) | -0.003 | (-0.028, 0.022) |
|  | Colombia | 0.039 | (-0.045, 0.123) | -0.039 | (-0.515, 0.437) | 0.121 | (-0.059, 0.301) |
| HR to HAB to Depression | Fixed across samples | 0.021 | (0.003, 0.039) | -0.016 | (-0.206, 0.174) | 0.012 | (-0.004, 0.028) |
| Fixed vs free test: | Naples | 0.008 | (-0.039, 0.055) | -0.119 | (-1.113, 0.875) | 0.057 | (-0.031, 0.145) |
| χ^2^[15]=17.508, *p*=0.289, | Rome | 0.029 | (-0.012, 0.070) | -0.555 | (-1.247, 0.137) | 0.041 | (-0.020, 0.102) |
| ΔRMSEA=-.001, | U.S. Black | 0.013 | (-0.012, 0.038) | -0.407 | (-0.917, 0.103) | 0.016 | (-0.015, 0.047) |
| ΔCFI=.002, | U.S. White | 0.043 | (0.008, 0.078) | -0.063 | (-0.455, 0.329) | 0.002 | (-0.033, 0.037) |
| ΔSRMR=.002 | U.S. Latinx | -0.003 | (-0.030, 0.024) | 0.163 | (-0.170, 0.496) | 0.006 | (-0.016, 0.028) |
|  | Colombia | 0.035 | (-0.041, 0.111) | 0.212 | (-0.202, 0.626) | 0.026 | (-0.076, 0.128) |
| HR to HAB to Anxiety | Fixed across samples | 0.022 | (0.004, 0.040) | -0.086 | (-0.288, 0.116) | 0.002 | (-0.014, 0.018) |
| Fixed vs free test: | Naples | 0.006 | (-0.037, 0.049) | -0.681 | (-1.679, 0.317) | 0.035 | (-0.034, 0.104) |
| χ^2^[15]=18.824, *p*=0.222 | Rome | 0.034 | (-0.009, 0.077) | -0.127 | (-0.662, 0.408) | -0.014 | (-0.063, 0.035) |
| ΔRMSEA=.001, | U.S. Black | 0.012 | (-0.010, 0.034) | -0.141 | (-0.753, 0.471) | -0.001 | (-0.030, 0.028) |
| ΔCFI=.002, | U.S. White | 0.046 | (0.011, 0.081) | -0.441 | (-0.958, 0.076) | -0.005 | (-0.044, 0.034) |
| ΔSRMR=.002 | U.S. Latinx | -0.006 | (-0.033, 0.021) | 0.081 | (-0.358, 0.520) | 0.014 | (-0.013, 0.041) |
|  | Colombia | 0.032 | (-0.039, 0.103) | 0.203 | (-0.162, 0.568) | -0.004 | (-0.080, 0.072) |

*Note:* HAB = hostile attribution bias; HR = hostile rumination. Change in model fit is calculated so that more positive models indicate that the free model performs better. For RMSEA and SRMR this is fixed – free; for CFI this is free – fixed.

# Table S7.

# *Multigroup analysis mediation coefficients for gender.*

|  |  | IV to Mediator (a) | | Mediator to DV (b) | | IV to DV (c’) | |
| --- | --- | --- | --- | --- | --- | --- | --- |
| Model | Group | b | 95% CI | b | 95% CI | b | 95% CI |
| HAB to HR to Aggression | Fixed across gender | 1.052 | (0.384, 1.720) | 0.047 | (0.006, 0.088) | -0.274 | (-0.456, -0.092) |
| Fixed vs free test: | Male | 0.769 | (-0.197, 1.735) | 0.041 | (-0.014, 0.096) | -0.222 | (-0.471, 0.027) |
| χ^2^[3]=0.89, *p*=0.828 | Female | 1.281 | (0.385, 2.177) | 0.056 | (-0.009, 0.121) | -0.326 | (-0.587, -0.065) |
| ΔRMSEA=-.001, |  |  |  |  |  |  |  |
| ΔCFI=-.001, |  |  |  |  |  |  |  |
| ΔSRMR=.001 |  |  |  |  |  |  |  |
| HAB to HR to Depression | Fixed across gender | 1.119 | (0.460, 1.778) | 0.023 | (-0.022, 0.068) | -0.184 | (-0.380, 0.012) |
| Fixed vs free test: | Male | 0.784 | (-0.157, 1.725) | 0.039 | (-0.016, 0.094) | -0.037 | (-0.268, 0.194) |
| χ^2^[3]=6.49, *p*=0.090 | Female | 1.326 | (0.452, 2.200) | -0.009 | (-0.093, 0.075) | -0.375 | (-0.698, -0.052) |
| ΔRMSEA=.001, |  |  |  |  |  |  |  |
| ΔCFI=.003, |  |  |  |  |  |  |  |
| ΔSRMR=.003 |  |  |  |  |  |  |  |
| HAB to HR to Anxiety | Fixed across gender | 1.178 | (0.492, 1.864) | 0.054 | (0.009, 0.099) | -0.447 | (-0.663, -0.231) |
| Fixed vs free test: | Male | 0.791 | (-0.193, 1.775) | 0.065 | (0.006, 0.124) | -0.402 | (-0.682, -0.122) |
| χ^2^[3]=1.723, *p*=0.632 | Female | 1.479 | (0.560, 2.398) | 0.040 | (-0.029, 0.109) | -0.472 | (-0.780, -0.164) |
| ΔRMSEA=-.001, |  |  |  |  |  |  |  |
| ΔCFI=-.001, |  |  |  |  |  |  |  |
| ΔSRMR=.002 |  |  |  |  |  |  |  |
| HR to HAB to Aggression | Fixed across gender | 0.058 | (0.025, 0.091) | -0.042 | (-0.246, 0.162) | 0.049 | (0.006, 0.092) |
| Fixed vs free test: | Male | 0.055 | (0.012, 0.098) | -0.159 | (-0.445, 0.127) | 0.035 | (-0.020, 0.090) |
| χ^2^[3]=3.041, *p*=0.385 | Female | 0.057 | (0.010, 0.104) | 0.091 | (-0.215, 0.397) | 0.070 | (0.005, 0.135) |
| ΔRMSEA=-.001, |  |  |  |  |  |  |  |
| ΔCFI=.000, |  |  |  |  |  |  |  |
| ΔSRMR=.001 |  |  |  |  |  |  |  |
| HR to HAB to Depression | Fixed across gender | 0.059 | (0.024, 0.094) | -0.058 | (-0.234, 0.118) | 0.040 | (0.003, 0.077) |
| Fixed vs free test: | Male | 0.056 | (0.011, 0.101) | 0.033 | (-0.167, 0.233) | 0.027 | (-0.014, 0.068) |
| χ^2^[3]=3.653, *p*=0.301 | Female | 0.060 | (0.009, 0.111) | -0.343 | (-0.715, 0.029) | 0.083 | (0.003, 0.163) |
| ΔRMSEA=.000, |  |  |  |  |  |  |  |
| ΔCFI=.000, |  |  |  |  |  |  |  |
| ΔSRMR=.002 |  |  |  |  |  |  |  |
| HR to HAB to Anxiety | Fixed across gender | 0.060 | (0.027, 0.093) | -0.168 | (-0.384, 0.048) | 0.028 | (-0.015, 0.071) |
| Fixed vs free test: | Male | 0.059 | (0.016, 0.102) | -0.145 | (-0.425, 0.135) | 0.040 | (-0.017, 0.097) |
| χ^2^[3]=0.753, *p*=0.861 | Female | 0.061 | (0.012, 0.110) | -0.197 | (-0.524, 0.130) | 0.009 | (-0.062, 0.080) |
| ΔRMSEA=-.001, |  |  |  |  |  |  |  |
| ΔCFI=-.001, |  |  |  |  |  |  |  |
| ΔSRMR=.000 |  |  |  |  |  |  |  |

*Note:* HAB = hostile attribution bias; HR = hostile rumination. Change in model fit is calculated so that more positive models indicate that the free model performs better. For RMSEA and SRMR this is fixed – free; for CFI this is free – fixed.

**A**

T2 HR

T3 Aggression

T1 HAB

T1 Aggression

.585**

0.03*

0.61**

-0.08*

0.63**

Indirect effect: *b* = 0.016, 95% bootstrap CI = (0.004, 0.035)

Total effect: *b* = -0.063, 95% bootstrap CI = (-0.135, 0.007)

**B**

T2 HR

T3 Depression

T1 HAB

T1 Depression

0.62**

0.01

0.56**

-0.27**

0.54**

Indirect effect: *b* = 0.008, 95% bootstrap CI = (-0.002, 0.24)

Total effect: *b* = -0.060, 95% bootstrap CI = (-0.136, 0.019)

**C**

T2 HR

T3 Anxiety

T1 HAB

T1 Anxiety

0.63**

0.03*

0.19

-0.24**

0.62**

Indirect effect: *b* = 0.02, 95% bootstrap CI = (0.005, 0.048)

Total effect: *b* = -0.22, 95% bootstrap CI = (-0.346, -0.105)

**D**

T3 HAB

T4 Aggression

T2 HR

T2 Aggression

.03**

0.01

0.04**

-0.03*

0.51**

Indirect effect: *b* = 0.000, 95% bootstrap CI = (-0.003, 0.004)

Total effect: *b* = 0.025, 95% bootstrap CI = (.002, .045)

**E**

T3 HAB

T4 Depression

T2 HR

T2 Depression

.03**

-.06

0.04

-0.08*

0.40**

Indirect effect: *b* = -0.002, 95% bootstrap CI = (-0.007, 0.002)

Total effect: *b* = 0.025, 95% bootstrap CI = (-0.001, 0.051)

**F**

T3 HAB

T4 Anxiety

T2 HR

T2 Anxiety

.03**

-.14

-.02

-0.08*

0.49**

Indirect effect: *b* = -0.005, 95% bootstrap CI = (-0.012, 0.000)

Total effect: *b* = 0.016, 95% bootstrap CI = (-0.019, 0.048)

*Figure S1.* Path analyses for mediational pathways from hostile attribution bias (HAB) through hostile rumination (HR) to outcomes (aggression, depressive symptoms, anxiety symptoms). Covariates (child age at time 1 and child gender) and parcels are included in all analyses but are not depicted. (A) Aggression outcome model. (B) Depressive symptoms outcome model. (C) Anxiety symptoms outcome model. Unstandardized coefficients are displayed. *= *p* < .05, ***p* < .005.
